# Novel germline *MSH2 *mutation in lynch syndrome patient surviving multiple cancers

**DOI:** 10.1186/1897-4287-10-1

**Published:** 2012-01-10

**Authors:** Ramunas Janavicius, Pavel Elsakov

**Affiliations:** 1 Department of Molecular and Regenerative Medicine; Hematology, Oncology and Transfusion Medicine Center, Vilnius University Hospital Santariskiu Clinics, Santariskiu st. 2, Vilnius LT-08661, Lithuania; 2 Institute of Oncology Vilnius university, Santariskiu st. 1, Vilnius LT-08660, Lithuania

**Keywords:** Lynch syndrome, *MSH2 *mutation, increased survival, multiple cancers, HNPCC

## Abstract

Lynch syndrome (LS) individuals are predisposed to a variety of cancers, most commonly colorectal, uterine, urinary tract, ovarian, small bowel, stomach and biliary tract cancers. The risk of extracolonic manifestations appears to be highest in *MSH2 *mutations carriers.

We present a carrier case with a novel *MSH2 *gene mutation that clearly demonstrates the broad extent of LS phenotypic expression and highlights several important clinical aspects. Current evidence suggests that colorectal tumors from LS patients tend to have better prognoses than their sporadic counterparts, however survival benefits for other cancers encountered in LS are unclear.

In this article we describe a family with a novel protein truncating mutation of c.2388delT in the *MSH2 *gene, particularly focusing on one individual carrier affected with multiple primary cancers who is surviving 25 years on. Our report of multiple primary tumors occurring in the 12-25 years interval might suggest these patients do not succumb to other extracolonic cancers, provided they are regularly followed-up.

## Introduction

Lynch syndrome (LS), or hereditary nonpolyposis colorectal cancer (HNPCC) syndrome, is genetically heterogeneous autosomal dominant disease, caused by mutations in one of at least four mismatch repair (MMR) genes, most frequently *MLH1 *or *MSH2*, which account for about 50% and 40% of cases respectively [1]. More than 1000 unique mutations were reported in each of these genes http://www.insight-group.org. Truncating mutations are the most frequent cause of deficient MMR function, comprising about 68% and 82% of the types of mutations found in the *MLH1 *and *MSH2 *genes respectively [1].

Clinically LS is characterized by a high risk for early-onset colorectal and endometrial cancers and also a moderate risk for urinary tract, ovarian, small bowel, stomach and biliary tract cancers. LS accounts for 2% to 5% of all colorectal cancers [2]. Recognition of LS, leading to molecular screening for MMR genes in a proband, is currently based on the clinico-familial Amsterdam II criteria and/or clinico-pathological Bethesda criteria [3]. Identification of LS causing mutations is important for clinical surveillance in carriers and genetic testing for at risk relatives.

Considerable variability exists in phenotypic expression and cancer risk among different gene mutations carriers. The risk of colon cancer appears to be greater in carriers of *MLH1 *vs other MMR genes, but the overall risk of all cancers (colonic and extracolonic) combined appears with *MSH2 *mutations [4]. In *MSH2 *mutation carriers, relevant genotype-phenotype correlations exist for benign and malignant sebaceous glands tumors (Muir-Torre syndrome) [2].

It is well known that colorectal tumors from LS patients tend to have better prognoses than sporadic colorectal tumors [5] and are associated with distinct histological characteristics such as tumor-infiltrating lymphocytes, mucinous/signet-ring differentiation and/or medullary growth patterns, as well as proximal localization [6]. However, the survival benefits for other cancers in LS are unclear.

There is some inconsistency among cases reported in the literature regarding the involvement of other tumors in LS, such as breast cancer [7], although breast cancer is not classically regarded a LS spectrum tumor [8].

In this article we describe a family with the novel protein truncating mutation of c.2388delT in the *MSH2 *gene, focusing particularly on one individual carrier affected with multiple primary cancers who is surviving 25 years on.

## Case report

**Figure 1:**
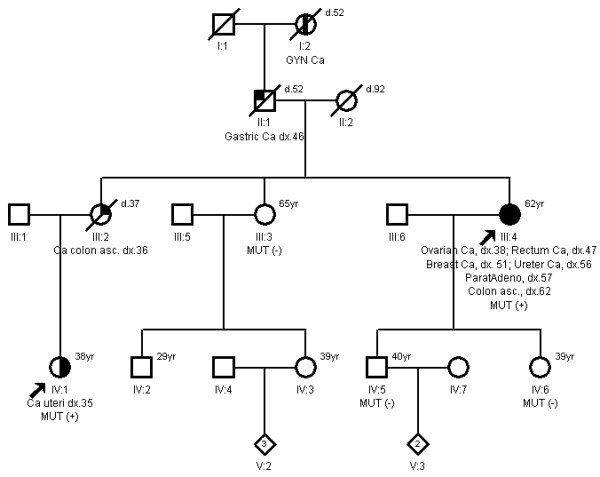
**Pedigree of the family with *MSH2 *c.2388delT mutation**.

Currently 63 year old index female patient (III:4) with a positive colorectal cancer family history was considered having LS after she presented with a colorectal cancer in her 47th year (1995), after she already had been diagnosed with ovarian cancer at the age of 38 (1986) (Figure 1). Her personal medical history revealed a total hysterectomy and bilateral-salpingoophorectomy as a consequence of treatment for ovarian cancer. The patient then conformed to Amsterdam II and Bethesda criteria [3]. For research purposes the patient was enrolled in a LS screening project and found to be a carrier of c.2388delT mutation in the 14 exon of *MSH2 *gene by direct sequencing. This genetic data was included in a collaborative study report of LS mutation spectrum from the Baltic countries and Poland [9], recently referenced in InSiGHT http://www.insight-group.org, although this mutation and phenotype were not characterized in more detail at the time.

The patient at the time of diagnosis received standard treatment in accordance with protocols established at the Lithuanian Institute of Oncology (briefly outlined in Table 1). After the rectal cancer diagnosis, she was followed-up by 1-2 yearly colonoscopies and clinical examinations as an outpatient.

**Table 1:** The development of multiple primary tumors in patient (individual - index III: 4).

Site of tumor	Age at diagnosis (years)	Histology	Stage (TNM)	Treatment
Ovary (right)	38	Mucinous cystadenocarcinoma	I (T1N0M0)	Radical hysterectomy+chemotherapy+ radiotherapy (1986)

Rectosigmoid	47	Mucinous adenocarcinoma	III (T3N1M0)	Resection of rectosigmoid part with "end-to-end anastomosis"+ chemotherapy

Breast (left)	51	Ductal adenocarcinoma	II (T2N0M0)	Mastectomy + radiotherapy (4 fields), tamoxifen (20 mg) 3 years

Ureter (left)	56	Transitional cell ureter carcinoma (G2)	II (T2N0M0)	Resection of ureter + nephroectomy

Adenoma of parathyroid	57			Ultrasound biopsy

Ureteral metastasis in liver (S5-6)	60	Transitional cell ureter carcinoma (G2)	IV	Radiofrequency ablation

Colon (ascending)	61	Adenocarcinoma with partial mucinous differentiation (G2)	II (T3N0M0)	Right hemicolectomy

All available pathology and medical reports of cancers occurring in this patient were reviewed.

During a prophylactic mammography performed at the age of 51, a tumor (3 × 2.5 cm) was localized in the lower-inner quadrant of the left breast and estrogen receptor positive infiltrative adenocarcinoma stage II (T2N0M0) was diagnosed. The patient underwent a left modified mastectomy, radiotherapy and received Tamoxifen treatment for 3 years. Chemotherapy was postponed due to persistent leucopenia and thrombocytopenia; the patient agreed to receive only one CMF (Cyclophosphamide, Methotrexate and 5-FU) regimen cycle.

After 5 years thereafter, at the age of 56, the patient arrived with complaints of left sided loin-flank pain. An enlarged ureter and left hydronephrosis were found and a moderately differentiated transitional cell carcinoma of the left ureter was confirmed histopathologically after partial resection. A left kidney nephrectomy was performed a few months later, since no kidney function was detected by excretional urography. Four years later an ureter carcinoma metastasis was suspected in the liver by CT scan, confirmed pathologically after liver mass biopsy and treated by percutaneous radiofrequency ablation.

Benign adenoma of the parathyroid gland was diagnosed by ultrasound guided biopsy at the age of 57.

At the age of 62, 1 year and 4 months after the last normal colonoscopy, the patient subsequently developed a stage II adonocarcinoma of the ascending colon (visualized endoscopically as a flat 2 cm lesion) and a right hemicolectomy was performed.

Retrospective immunohistochemical analysis of MMR proteins in the patients colorectal cancer and urinary tract carcinoma showed a consistent loss of expression of MSH2/MSH6 protein complexes.

**Figure 2:**
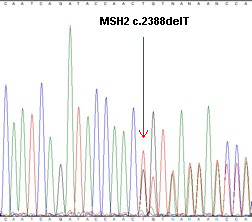
**Sequence analysis of *MSH2 *gene (Genebank accession number U04045) with c.2388delT (p.Thr796ThrfsX15) in exon 14**.

The niece of the index patient (IV:1, 38 years) was independently referred to the cancer geneticist in 2010 due to a cancer family history and endometrial cancer diagnosis at the age of 35 years (Figure 1), meeting both Amsterdam II and Bethesda criteria. Analysis of mononucleotide BAT25, BAT26 and CAT25 markers [10] showed instability of all of them and immunohistochemistry of MLH1/PMS2 and MSH2/MSH6 proteins indicated the loss of MSH2/MSH6 expression in the endometrial tumor; direct sequencing of the *MSH2 *gene on ABI 3500 (Applied Biosytems) genetic analyzer revealed the same c.2388delT mutation (p.Thr796ThrfxX15) in exon 14 (Figure 2). This frame shifting mutation is predicted to induce a premature "stop" codon at the downstream 811 amino acid level, which results in truncated MSH2 protein, presumably further degraded by nonsense mediated decay (NMD) [11]. This mutation has not been reported in detail in the literature.

Neither sister (III:3) nor children (IV:5, IV:6) of the proband were found to be carriers of the familial deleterious *MSH2 *mutation.

## Discussion

LS can be difficult to diagnose, due to a lack of specific phenotypic features, and the need for a high level of clinical suspicion when encountering cardinal LS features where extracolonic cancers are integral to the syndrome [6]. Our presented carrier case with a novel *MSH2 *gene mutation clearly demonstrates the broad extent of LS phenotypic expression and highlights several important clinical aspects.

Firstly, the patient over time presented with five primary cancers (in different organs) all of them, except that of breast, typical for the tumor spectrum of LS. Early-stage ovarian mucinous carcinoma was the first manifestation of LS spectrum in the proband. The lifetime risk of ovarian cancer in Lynch syndrome is approximately 10-12% [12], with higher risks (36%) reported in *MSH2 *carriers [13]. Recent estimates of age-specific cumulative cancer risks in Lynch syndrome families suggest lower than published elsewhere ovarian cancers risk (24% ovarian cancer risk in *MSH2*) [14]. MMR-deficient ovarian cancers are biologically and clinically different from *BRCA *deficient cancers, the former overrepresented by non-serous histology types, early-stage and early-onset disease [12,15].

For LS women, the lifetime risk for endometrial cancer (40-80%) is substantially higher than that for colorectal cancer (30-60%) [12]; the risk being greater in *MSH2 *and *MSH6 *carriers [16]. We assume that the increased occurrence of endometrial cancer in the proband III:4 was eliminated by radical hysterectomy performed after the diagnosis of ovarian cancer at the age of 38, although the other family member IV:1 developed endometrial cancer at an earlier age (35 years).

The majority of colonic cancers seen in LS patients have a proximal predilection for site of occurrence and affect the right colon [6], while rectal cancer (left-sided) is seen in ~20% of *MLH1 *and *MSH2 *carriers [2]. Our patient presented with firstly left sided then later right sided colonic cancer. Our patient's CRCs showed typical histopathological features of LS: mucinous histology and lymphocytic infiltration. It was observed that patients with an initial left-sided CRC developed a metachronous colon tumor in a statistically significant shorter time span (medium 12 years) than patients with a first right-sided CRC (medium 23 years) [2]. Furthermore, one third of patients having had surgery for a LS-associated CRC will have a second primary CRC within 10 years of surgical treatment if the surgery was less than a subtotal colectomy [6]. Consistently, our patient had developed right-sided metachronous CRC 14 years after her first presentation for rectal cancer.

Cancers of the upper urinary tract (renal pelvis/ureter) occur more frequently in LS, with a lifetime risk of 4% compared with < 1% in the general population [17], and are more common in *MSH2 *mutation carriers, reaching up to 9% for women and 20% for men who are such mutation carriers [18,19]. Upper urinary tract cancers are rarely diagnosed before the age of 40 years [2]. In our proband carcinoma of the ureter was the most aggressive cancer type, unfortunately metastasizing to the liver - a poor prognostic factor.

Breast cancer is not generally regarded as a tumor specific to LS, and lifetime risks are not increased in LS patients, although MMR may have effects on the progression of breast tumors [7,16]. Of note, is that the occurrence of both ovarian and breast cancer in our patient led to an erroneous assumption of the involvement of *BRCA *mutations by one physician. We have additionally screened (by high resolution melting analysis) *BRCA1/2 *genes for five common mutations in Lithuania (4 in *BRCA1 *and 1 in *BRCA2*). On the other hand, mucinous ovarian cancer is not found to be associated with *BRCA1/2 *genes [12,15]. About half of breast cancers arising in MMR gene mutation carriers have MMR proteins deficiencies and thus breast tissue samples may be used to improve identification of patients at risk [7,20]. Unfortunatelly, we were unable to perform additional MMR immunohistochemical staining because of the unavailability of necessary material.

We were unable to confirm neither the history nor find appearance of sebaceous tumors (Muirr-Torre variant), which can be seen in *MSH2 *or *MLH1 *carriers.

Additionally, the most striking feature of our case report is that our *MSH2 *mutation carrier, who experienced multiple cancers, is surviving 25 years after her first cancer diagnosis. Improved survival with CRC, compared to unselected sporadic CRC cases, has been consistently reported in LS patients [21,22], although this feature is also common for non-syndromic MSI instable CRCs. The data for other extracolonic LS cancers are scarce, and one study found no survival rate differences between ovarian cancer patients with LS and those with sporadic ovarian cancers [23]. Our survival data for CRC strongly supports increased survival in LS/MMR defective cases and sharply contrasts with previous observations for ovarian cancer. A very recent study found that the risk of dying from (diagnosed) ovarian cancer in germline MMR deficient patients is about 2% [24]. Our results are in line with the notion that MMR genes may predispose to a biologically different type of ovarian cancer than *BRCA1/2 *or in the general population, characterized by early stage disease at presentation and favorable prognosis [24]. After the *MSH2 *mutation confirmation in 1998 (at the age of 50), our case was regarded consistent with LS and therefore 12 years of survival data were collected prospectively. Given the absence of published survival data for extracolonic cancers in LS patients, our report of multiple primary tumors occurring in the 12-25 years interval might suggest that these patients do not succumb to other extracolonic cancers, provided they are on regularly follow-up.

Currently, only colonoscopic surveillance is proven to reduce the incidence of mortality from CRC and so is recommended every 1-2 years beginning at the age of 20-25 years [5]. Our case demonstrates that an optimal colonoscopy interval lies closer to 1 year, since a second asymptomatic stage II CRC was diagnosed only 1 year and 4 months after the last normal colonoscopy.

The other LS patient (IV:1) was recommended to be followed up by annual colonoscopy, abdominal ultrasound, urinalysis, gastroduodenoscopy and *H. pylori *detection and eradication. However, effective surveillance protocols for other extracolonic cancers need still to be identified [19].

In conclusion, prolonged survival in our case of a carrier of the novel *MSH2 *mutation affected by multiple cancers, raises current challenges in the management of individuals with LS. Obviously, from a single case we can not unequivocally draw conclusions about multiple tumor phenotype correlations with the particular mutation, without having to admit possible influences of modifier genes [25], which may also have effects on tumor spectra to be encountered and survival rates of probands. Additionally, host immune system responses might be enchanced to MMR-deficient tumors [26].

## Consent

Written informed consent was obtained from the patient for publication of this case report. A copy of the written consent is available for review by the Editor-in-Chief of this journal.

## Competing interests

The authors declare that they have no competing interests.

## Authors' contributions

RJ contributed to the genetic counselling of the familly, molecular genetic testing and data analysis, drafted and reviewed the manuscript; PE provided colonoscopic surveillance for the familly members, collected clinical data and drafted the manuscript. All authors read and approved the final manuscript.
